# Can Graft vs. Leukemia Effect Be Uncoupled From Graft vs. Host Disease? An Examination of Proportions

**DOI:** 10.3389/fimmu.2020.00777

**Published:** 2020-04-28

**Authors:** Elizabeth Krieger, Amir Ahmed Toor

**Affiliations:** ^1^Department of Pediatrics, Virginia Commonwealth University, Richmond, VA, United States; ^2^Department of Internal Medicine, Massey Cancer Center, Virginia Commonwealth University, Richmond, VA, United States

**Keywords:** hematopoietic cell transplantation, graft vs. host disease, graft vs. leukemia effect, whole exome sequencing, tumor vaccination

Hematopoietic cell transplantation (HCT) provides potentially curative therapy for high-risk hematological malignancies, predominantly through alloreactivity mediated by donor immune effectors directed at a recipient's malignant cells; this is termed graft vs. leukemia (GVL) effect (1). This beneficial effect has historically been associated with a similar donor immune attack on normal recipient tissues, graft vs. host disease (GVHD) (2). At this time both of these entities, GVL and GVHD are considered to be stochastically determined, i.e., prior to transplant one cannot reliably determine which patient will develop one or both outcomes. The high complexity of the system at hand which includes patients with different malignancies, varying human leukocyte antigen (HLA) types, and different immune effectors involved in these processes has meant that logic-based therapeutic choices which impact variables associated with GVL are studied by determining the probability of the desired clinical outcomes in large populations of patients. Such studies have allowed an incremental improvement in the clinical outcomes of recipients of similarly HLA matched donor HCT. The introduction of high-resolution HLA matching and HLA DPB1 matching were both such incremental changes which helped improve survival in recipients from HLA matched HSC donors (3–5). Despite these advances, the apparent randomness in the potential for developing alloreactivity remains. This apparent randomness derives, in part from these phenomena having their origin at a molecular level, with the recognition of minor histocompatibility antigens (mHA) and tumor specific antigens (TSA) bound to HLA molecules on the antigen presenting cells (APC), by unique T cell receptors (TCR) on T cell clones. This recognition triggers T cell responses which effect the observed clinical outcomes.

To develop a deeper understanding of the alloreactive processes governing the relative balance of GVHD and GVL one has to understand the antigenic landscape at hand in a HCT recipient at the molecular level. Herein is presented a model which examines the relative difference in the genetic *potential* for developing either GVL, using tumor specific antigen (TSA) burden, or likelihood of developing GVHD, using minor histocompatibility antigens (mHA). Historically haematopoietically restricted mHA (6–8), cancer testis antigens (9), protein splice variants (10) and in some instances even retroviral elements (11) have been implicated in producing GVL effects, with some of these elements also contributing to GVHD. While haematopoietically restricted mHA have been implicated in the development of GVHD and protection from relapse (12), HLA presentation is a prerequisite for this to occur (13). Thus, far ~60 haematopoietically restricted minor histocompatibility antigens have been described with antigen presentation restricted to a limited spectrum of HLA allotypes, precluding broad utility in patients (7). Thus, to optimize clinical outcomes, it is imperative to develop methodology which will allow personalized computation of the probability of GVHD or GVL developing in unique HCT donor-recipient pairs.

Hematological malignancies are driven by DNA mutations which develop in normal cells over time as a result of exposure to external mutagens and intrinsic processes, such as errors in DNA replication (14). The mutational burden of adult cancers ranges widely; for example, solid tumors may average from 33 to 66 somatic mutations which alter their protein structure and function. Cancers such as, melanoma and lung cancer are on an extreme end of this spectrum, possessing ~200 mutations per tumor, and thus are susceptible to immune therapy (15). On the opposite end of this spectrum, hematologic malignancies have some of the lowest mutational burdens, with leukemias harboring ~9.6 mutations per tumor (14, 16–20). Mutated genes expressed in these tumors may be recognized as non-self-proteins by the immune system, and targeted by the GVL mechanism (21).

Point mutations were first shown to induce a naturally occurring T cell response in a patient with melanoma (22). However, initial studies of cancer immunotherapy were hampered by technological challenges encountered in deriving patient specific TSA libraries. In the past decade, next generation sequencing (NGS) or “deep” sequencing has allowed the sequencing of thousands of small fragments of DNA in parallel, such that an entire genome may be rapidly sequenced (23). NGS has allowed cataloging of the entire library of potential TSA in a variety of human malignancies. The full impact of this knowledge of individualized genetic profiling of cancers was first observed when utilizing programed death receptor PD-1 and programed death-ligand (PDL) receptor inhibitors. Check point blockade allows unimpeded autologous TSA specific T cell mediated killing, which is most significant in tumors with a higher mutational burden, as there are theoretically more TSA presented on MCH class I and class II molecules with a greater mutational burden (14, 24, 25).

In contrast, the relatively low mutational burden of hematologic malignancies does not meet the thereshold necessary to effectively utilize immunotherapy and PD1/PDL1 blockade (26, 27). Common hematologic driver mutations including NPM1 which are expressed in 30–35% of cases of AML have been shown to be expressed by AML blasts and may be targeted by TCR gene transfer (28). Several other specific mutations including BCR-ABL, WT1, and PR1 have also been shown to effect outcomes after HCT (29–31). Nevertheless, such unique mutations are usually not adequate to generate an intrinsic GVL response in the vast majority of patients. Despite this relative dearth of tumor associated neo-antigens, hematological malignancies have proven to be susceptible to the GVL effect of an allograft, some times without GVHD developing (32–34). One may therefore ask, is it possible to apply NGS to the transplant setting in order to understand how one may uncouple GVL from GVHD in the majority of patients? This goal has been sought by many a group who have tried to better predict GVHD and GVL by examining biomarkers (35), cytokines (36, 37), mass spectrometry data (38), natural killer cell markers (39). Modification of the conditioning and GVHD prophylaxis regimens have also been attempted to accomplish the dissociation of GVHD from GVL (40–43). However, while all of these factors play important roles in the GVHD and GVL phenomenon, if both at their core are centered on peptide presentation and immune attack, it is not likely that we can always dissociate GVL from GVHD.

A computational approach may be taken to develop a partial understanding of the GVHD-GVL balance in HLA matched HCT. As stated above, on average hematologic malignancies contain ~10 protein coding, exomic mutations which may be immunogenic. For patients with these and other TSA resulting from mutations, logically in each individual, the number of tumor specific peptide antigens presented will then depend on their HLA type, the specific mutations and the spectrum of mutated peptides presented by those HLA molecules. As an example, a study of over 600 patients with multiple myeloma showed an average of 64 nonsynonymous mutations. Neoantigen load was then predicted *in silico* by identifying mutant peptides predicted to bind class I HLA molecules. Predicted neoantigen were defined as any unique peptide: HLA combination with mutant binding affinity IC50 less then 500 nM. This revealed the average predicted neoantigens to be 23 in number, with 9 expressed neoantigens. This outlines the fact that not all neoantigens are either expressed or presented on HLA (17). This number then gives an approximate estimate of the isolated GVL inducing potential for multiple myeloma. However, the average number of nonsynonymous mutations in leukemia is typically much lower, as noted above. If we were to extrapolate using the ratio of 64 nonsynonymous mutations to its 9 expressed neoantigens, one could predict that hypothetically hematologic malignancy on average would be unlikely to express >10 neoantigens. In actual fact the true number of TSA will vary with each individual based on the number of nonsynonymous mutations present, type of mutation (i.e., point vs. frame shift mutations) their antigenicity, cleavage potential of the proteins harboring the mutations, the HLA binding affinities of the mutant peptides and the HLA type in an individual, among other factors. While, this may underestimate of the expressed neoantigens burden of hematologic malignancy, a study of antigen presentation of multiple malignancy types including hematologic malignancies and solid tumors indicated that there are ~1.5 expressed neoantigens per point mutation and 4 per frameshift mutation (44), suggesting that the estimate presented here is not too far from reality.

This may hold true even if one considers other TSA sources that may contribute to GVL, including those derived from normally repressed proteins such as cancer testis antigens. These are antigens normal expressed in “immunologically privileged sites” such as, testicular or trophoblastic tissues, and are thus immunogenic. When expressed, these will offer a potential GVL target, which will not be dependent on TSA, and will add to the TSA burden. However, there is variability introduced at the response end of this cascade, since some of these mutations may lead to too strong a TCR affinity and down regulation by central tolerance, while others with a more optimal affinity being allowed to escape central tolerance while still allowing allowreactivity (45). All in all, it is unlikely that most hematological malignancies have a very large abundance of TSA to drive an isolated GVL phenomenon.

With an estimate of the TSA in hematological malignancies established, one may next attempt to determine how likely it is to unravel GVL from GVHD. NGS also offers a perspective into the genetic background of GVHD alloreactivity. Exome sequencing in both hematopoietic stem cell as well as solid organ transplant recipients has demonstrated a vast library of potential mHA which provide an alternative set of targets for donor T cells. Whole exome sequencing (WES) of transplant donors and recipients was performed in a group of HLA matched donors and recipients, and demonstrated an average of >6,000 non-synonymous single nucleotide polymorphisms (SNP) per HLA matched donor-recipient pair (DRP) (46). These polymorphisms when translated into peptide sequences *in silico*, yielded an average of 2,254 peptides/DRP with the potential to bind HLA-A, -B and -C molecules with intermediate to high affinity (IC50 of <50 nM, NetMHCpan ver2.0) (47) and represented an alloreactivity potential for a given HSCT DRP. The SNPs when compared to the mutations used to estimate TSA, are much larger in number, indicating that mHA may provide the dominant antigen background in terms of generating alloreactivity following HCT. Similar data regarding the extent of genomic variation between transplant donors and recipients have been reported by other groups investigating genomic variation in transplant recipients, in both solid organ transplants (48) and in HCT (49–53), as well as in models predicting GVL specific libraries (54). This abundance of SNPs across the exome in unique HCT donor-recipient pairs is an eye-opening finding compared to the average 10 mutations per hematologic neoplasm. This relative antigen abundance of potential mHA compared to the potential TSA estimate is graphically depicted to scale in Figure 1.

**Figure 1:**
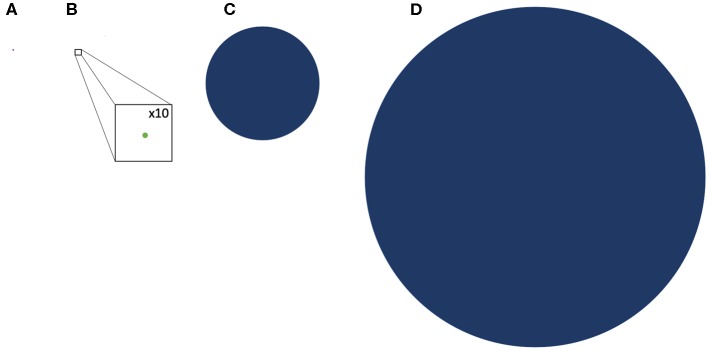
Comparative representation of the TSA and GVL potential of hematologic malignancy and GVHD drawn to scale. **(A)** representation of the average mutational burden of hematologic malignancy (9 SNPs) **(B)** A representation of the TSA, the potential peptide presentation of hematologic malignancy mutations in HLA class l; GVL Burden (drawn with x10 magnification to make visible) **(C)** representation of the potential DRP minor histocompatibility antigen proteins loaded onto HLA class l; GVHD burden **(D)** representation of the average SNP burden between DRP resulting from nonsynonymous polymorphisms.

While the sheer number of mHA alone vastly outnumbers the potential TSA in hematologic malignancy, these numbers do not tell the whole story. Whether the potential mHA result in a T cell proliferation depends on several factors, such as peptide cleavage potential, antigen binding affinity, and critically, T cell clones bearing receptors that might recognize the mHA-HLA complexes. Crucially, the T cell receptor affinity for HLA-mHA or HLA-TSA complexes also needs to be adequate to ensure T cell engagement and activation. Mathematical modeling of T cell expansion in response to these HLA-antigen complexes has given important insights into the quantitative principles at hand in these processes. First, the expansion of donor T cells recognizing specific antigens will be proportional to the amount of antigen available, i.e., the expression level of the antigen bearing protein will determine the extent of T cell expansion. Secondly, this T cell expansion is likely governed by the affinity of the antigen to the HLA molecule, and the affinity of the T cell receptors for antigen-HLA complex. This is an exponential relationship, with T cell growth increasing non-linearly in response to changing affinity. An important clue to this is provided by the T cell clonal frequency distribution which follows Power Law when these are plotted out for T cell clones present in normal individuals (55).

$$\begin{array}{l} T\;cell\;frequency\propto Antigen\;expression\times e^{mHA-HLA\;affinity\;\times \;TCR\;affinity} \end{array}$$

Based on the above model, an alloreactive donor cytotoxic T cell response was simulated. To do this the array of mHA in each patient was considered as an operator matrix modifying a hypothetical cytotoxic T cell clonal vector matrix. Utilizing the basic assumption that T cell expansion will be governed by the binding affinity of the variant peptide to HLA, and for model estimation of antigen driven T cell proliferation, assuming unit affinity of the TCR for each mHA-HLA complex (since this was not known for this particular set of antigens), each responding T cell clone's proliferation was determined by the logistic equation of growth (Figure 2). Assuming uniform growth conditions, *r* values in the logistic equation, these simulations, showed that the *simulated* organ-specific alloreactive T cell clonal growth had marked variability, with orders of magnitude of difference between different HLA matched DRPs (*N* = 78). This was because of the differences in the unique polymorphic peptide sequences and their binding to the many different HLA types. In this study higher total and organ-specific T cell counts were associated with the incidence of moderate to severe GVHD (56). T cell growth in these simulations exhibited a sigmoid, logistic dynamic over time similar to immune reconstitution kinetics exhibited by allograft recipients (57). This model predicted the emergence of a limited number of dominant T cell clones responding to highly expressed and high affinity mHA—HLA class I complexes unique to each individual depending on their HLA type. On the other hand, there was a large number of low frequency clones responding to poorly expressed protein-derived mHA, weakly bound to the corresponding HLA. When the model was adjusted to incorporate competition with dominant higher affinity clones, it demonstrated chaotic dynamics with suppression of the lower affinity clones in early time points, identifying this as a possible contributor to the stochasticity observed in the clinical setting. Further, once variability in TCR affinity for the mHA-HLA complexes is accounted for in this model, then the even greater variability and randomness in T cell responses may be observed between different donor-recipient pairs. Change in the term for growth rate, *r* in the model will have profound impact on the variability seen and GVHD risk. When evaluated for HLA class II molecule presentation, these alloreactive mHA libraries further expanded several-fold given the longer peptide sequences which may bind HLA class II molecules, increasing the mathematical complexity at hand. Nevertheless, this work demonstrates that these antigen arrays are susceptible to mathematical modeling and thus of potential use in estimating the likelihood of GVHD occurring in HLA matched (or mismatched) SCT DRP (58). Such estimates will potentially serve to personalize GVHD prophylaxis regimens to allow optimal GVL effect in future trials, while suppressing GVHD.

**Figure 2:**
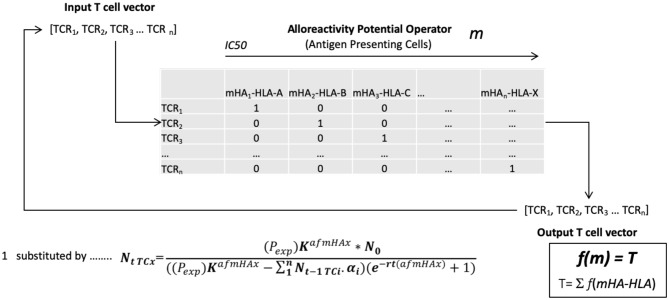
The vector operator model of minor histocompatibility antigen presentation to T cell receptors in an individual. TCR_i_- i^th^ T cell receptor; mHA_i_-HLA- i^th^ minor histocompatibility antigen-HLA complex; Nt_TCx_- Number of T cell at time t; P_exp_- polymorphic protein expression; K- Growth constant; N_0_- Starting T cell count; afmHA- Affinity of mHA*TCR affinity; *r*- Growth rate; *m*- cumulative mHA-HLA burden, alloreactivity operator; *T*- Total simulated T cell vector.

With these data in mind, when the relative number of tumor specific antigens and minor histocompatibility antigens are examined it becomes obvious that the relatively small number of TSA compared with mHA, may in most individuals result in outcompeting of tumor specific targets, by normal tissue targets setting up the field for GVL occurring in the company of GVHD (Figure 1). Thus, polymorphic normal recipient antigens (mHA) expressed in the malignant clones will be more likely to be presented to the donor T cells and contribute to a relapse-free-state, than TSA. The mathematics are further complicated by the possibility that the TSA compete not only with the mHA for presentation, but also with the non-polymorphic/non-antigenic peptides in the recipient's tissues, which will far outnumber both these sets of peptides, since these will also be loaded onto the HLA molecules and presented to the donor helper and cytotoxic T cells. The mathematics dealing with this problem were introduced in the paper by Salman et al. It is also imperative that the immunogenic antigens have peptides with an affinity to both HLA class I and HLA class II molecules and be expressed in a particular malignancy in an individual for those to be effective at provoking an immune response.

All is not lost in the mathematical medley of chaos and combinatorics. It is clear that the quantitatively driven T cell responses depend on relative antigen abundance and HLA affinity. Traditionally HSCT is done with patients in remission, and as immunosuppression is withdrawn, they may develop chronic GVHD, which confers protection from relapse, and in a few patients GRFS might be observed. This likely depends on both the extent of T cell clonal diversity emerging after transplantation, as well as the balance of antigen expression. It is therefore critical to understand the notion of relative antigen abundance (Figure 3), such that to elicit an effective immune response an antigen has to be present in an adequate quantity. Such relative antigen abundance of TSA and mHA may be modulated by vaccination using TSA, as has been reported in melanoma patients (59). This may increase the likelihood of GVL developing in a GVHD-free state in patients with hematological malignancies. It is important to recognize the logistic growth kinetics of T cell clones with an early exponential growth phase, and the importance of timing in vaccine administration before the onset of this growth. Another approach already in practice is to use hypomethylating agents to alter the expression of immunogenic cancer testis antigens (9). This therapy provides an extensive library of alternative immune targets for the donor T cells to focus on and has been successfully combined with donor lymphocyte infusions to treat post allograft relapse (60). It is to be recognized that this model only partially encompasses the complexity of normal and post-transplant immune responses and does not give a complete explanation for the GVHD-GVL dissociation observed in patients who experience GRFS, That state represents a complex interplay of the factors described here with conditioning regimens and GVHD prophylaxis, and of course tumor growth kinetics. Antigen presentation triggered by tissue injury and cytokine release are critical factors in these calculations, as are pharmacological suppression of T cell growth, and elimination of T cell clones.

**Figure 3:**
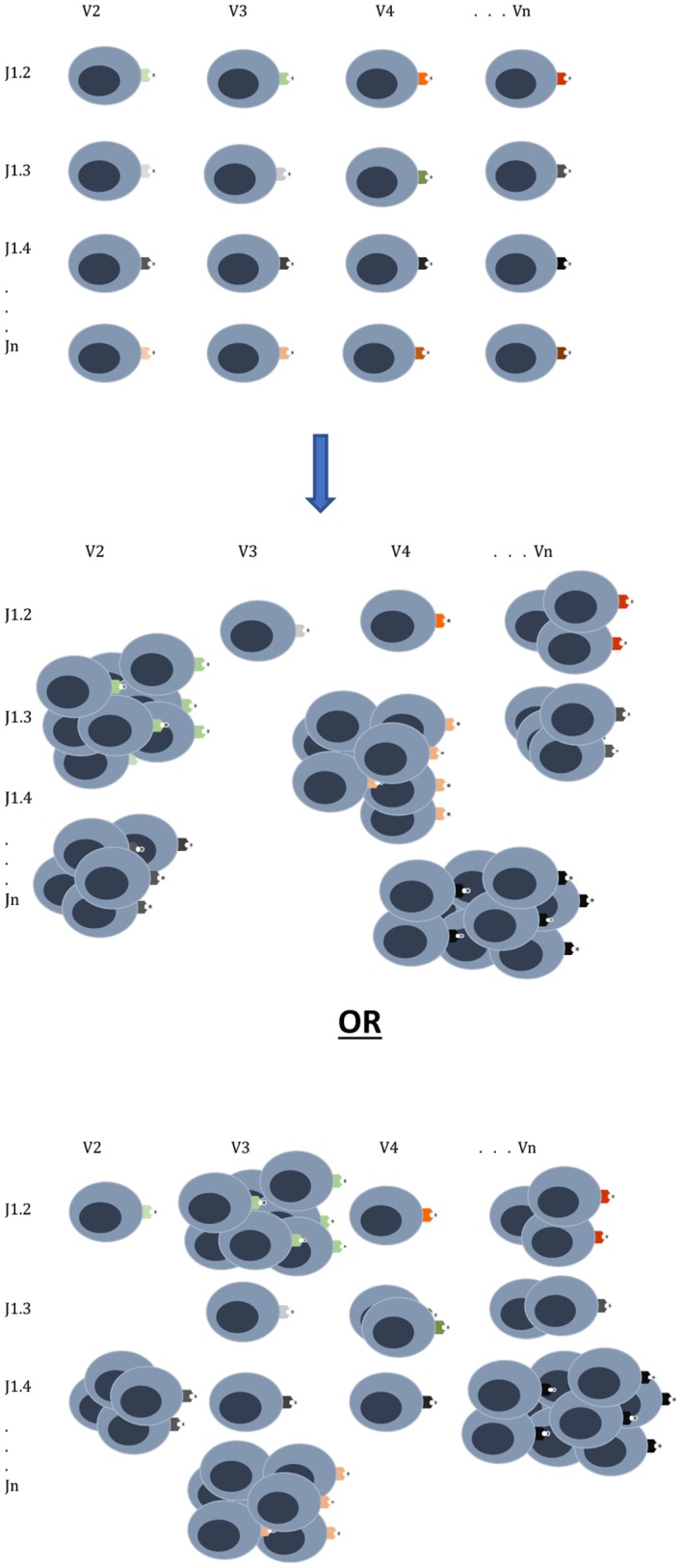
T cell clonal proliferation will depend on affinity and abundance of antigen at the time of initial exponential expansion. The donor graft has a T cell clonal repertoire with the potential to react to many different antigens. Once infused into the recipient the T cell clones expand in proportion to the relative antigen affinity and abundance as can be seen in two different scenarios emerging from the same donor cell infusion. Other factors which will influence this growth are cytokines, degree of tissue injury and pharmacotherapy for GVHD prophylaxis.

In conclusion, mathematical modeling of immune reconstitution, guided by NGS, along with an in-depth analysis of the relative expansion of donor T cell clones in response to the differentially expressed TSA and normal recipient antigens in individual patients, may allow a deeper understanding of the apparently stochastic nature of clinical outcomes observed at a population level. Mathematical modeling of T cell responses has revealed the chaotic dynamics of post-transplant immune responses, when multiple antigens with different HLA binding affinities and tissue expression levels are studied (58, 61, 62). Thus, stochasticity is built into the system, however, the probability windows for GVHD-GVL determination, may be narrowed by a using tools such as NGS of normal and malignant recipient, as well as donor exomes, and mathematical simulation of alloreactive T cell responses to mHA and TSA. These strategies can be used to identify the optimal TSA which would yield a T cell response, and these may then be used to derive tumor specific vaccines, altering the relative antigen abundance at crucial early times following SCT. Thus, in-depth genomic analysis may eventually allow us to truly develop precision medicine tools for optimizing patient outcomes following SCT.

## Author Contributions

EK and AT developed the idea and wrote the manuscript.

## Conflict of Interest

The authors declare that the research was conducted in the absence of any commercial or financial relationships that could be construed as a potential conflict of interest.
